# Supplementary material for: Using Dynamic Oral Dosing of Rifapentine and Rifabutin to Simulate Exposure Profiles of Long-Acting Formulations in a Mouse Model of Tuberculosis Preventive Therapy
Source: Antimicrob Agents Chemother. 2023 Jun 14;67(7):e00481-23. doi: 10.1128/aac.00481-23 (PMC10353356; doi:10.1128/aac.00481-23)
Supplement: Supplemental file 1 — Supplemental material. Download aac.00481-23-s0001.pdf, PDF file, 2.7 MB [file aac.00481-23-s0001.pdf]

Using dynamic oral dosing of rifapentine and rifabutin to simulate exposure profiles of long-acting formulations in a mouse model of tuberculosis preventive therapy

YS Chang, SY Li, H Pertinez, F Betoudji, J Lee, SP Rannard, A Owen, EL Nuermberger, NC Ammerman

Online Supplemental Materials

Figures

- **Fig. S1.** Dose linearity of rifapentine and rifabutin in uninfected female BALB/c mice.
- **Fig. S2.** Dosing simulations for rifapentine and rifabutin to achieve indicated target plasma concentrations in the first PK/PD study.
- **Fig. S3.** Simulated and observed rifapentine plasma concentrations and PK parameters from the first PK/PD study.
- **Fig. S4.** PK/PD relationships of rifapentine and rifabutin in the first PK/PD study.
- **Fig. S5.** Simulated and observed rifabutin plasma concentrations and PK parameters from the first PK/PD study.
- **Fig. S6.** Exposure profiles and dosing simulations for rifapentine in the second PK/PD study.
- **Fig. S7.** Rifabutin exposure profiles and dosing simulations, and observed exposures in the second PK/PD study.

Tables

- **Table S1.** MIC/MBC data for rifampin, rifapentine, and rifabutin against *M. tuberculosis* H37Rv.
- **Table S2.** PK parameters associated with oral dosing of rifapentine and rifabutin in uninfected male BALB/c mice.
- **Table S3.** Original and final experiment schemes for the first PK/PD study evaluating rifapentine and rifabutin regimens designed to maintain stable, predefined target plasma drug concentrations.
- **Table S4.** Summary of *M. tuberculosis* H37Rv lung CFU counts in first PK/PD study.
- **Table S5.** Oral dosing regimens designed to simulate LAI exposures of rifapentine and rifabutin in the second PK/PD study.
- **Table S6.** Experiment scheme for the second PK/PD study evaluating simulated LAI regimens of orally dosed rifapentine and rifabutin.
- **Table S7.** Summary of *M. tuberculosis* H37Rv lung CFU counts in second PK/PD study.
- **Table S8.** Description and CFU data for bacterial suspensions used for aerosol infections of mice.
- **Table S9.** Summary of *M. bovis* rBCG30 lung CFU counts in first PK/PD study.
- **Table S10.** Summary of *M. bovis* rBCG30 lung CFU counts in the second PK/PD study.

Data Files

- **Data File S1.** Individual mouse PK data from PK study in female and male BALB/c mice.
    - **S1.1.** Individual mouse PK data from rifapentine-dosed female BALB/c mice.
    - **S1.2.** Individual mouse PK data from rifabutin-dosed female BALB/c mice.
    - **S1.3.** Individual mouse PK data from rifapentine-dosed male BALB/c mice.
    - **S1.4.** Individual mouse PK data from rifabutin-dosed male BALB/c mice
- **Data File S2.** Individual mouse CFU and PK data from the first PK/PD study.
    - **S2.1.** Individual mouse CFU data, Week -19 time point (rBCG30 immunization).
    - **S2.2.** Individual mouse CFU data, Week -13 time point (first *M. tuberculosis* challenge infection).
    - **S2.3.** Individual mouse CFU data, Week -7 time point (second *M. tuberculosis* challenge infection).
    - **S2.4.** Individual mouse CFU data, Day 0 time point (day of treatment initiation).
    - **S2.5.** Individual mouse CFU data, Week 3 time point.
    - **S2.6.** Individual mouse CFU data, Week 3 + 1.5 days time point.
    - **S2.7.** Two-way ANOVA of CFU data at Week 3 and Week 3 + 1.5 days.
    - **S2.8.** Individual mouse PK data.
- **Data File S3.** Individual mouse CFU and PK data from the second PK/PD study. D study.
    - **S3.1.** Individual mouse CFU data, Week -12 time point (rBCG30 immunization).
    - **S3.2.** Individual mouse CFU data, Week -6 time point (*M. tuberculosis* challenge infection).
    - **S3.3.** Individual mouse CFU data, Day 0 time point (day of treatment initiation).
    - **S3.4**. Individual mouse CFU data, Week 2 time point.
    - **S3.5.** Individual mouse CFU data, Week 4 time point.
    - **S3.6.** Individual mouse CFU data, Week 8 time point.
    - **S3.7.** Two-way ANOVA of CFU data.
    - **S3.8**. Individual mouse PK data.

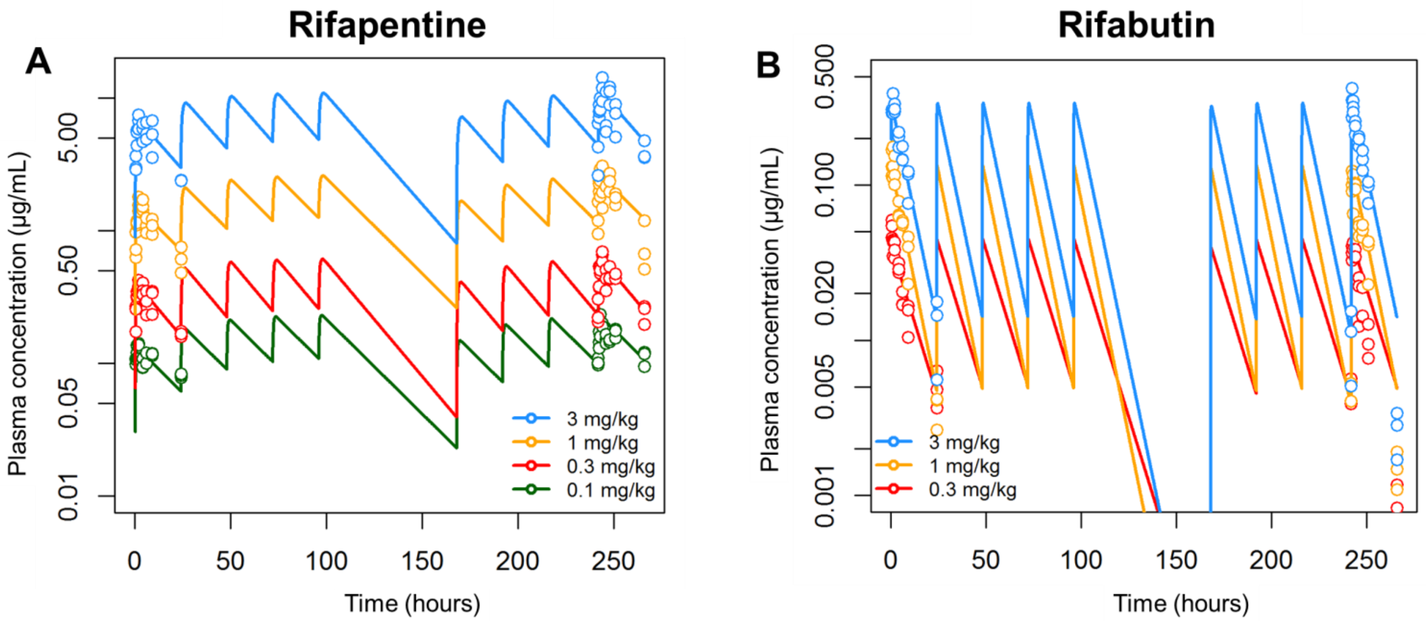

Fig. S1. Dose linearity of rifapentine (A) and rifabutin (B) in uninfected female BALB/c mice. Data points represent the observed plasma concentrations of individual samples, and the lines indicate the modeled PK curves fitted to the data. All regimens were orally dosed once daily, five days per week (Monday-Friday). All individual mouse PK data are provided in **Data File S1**.

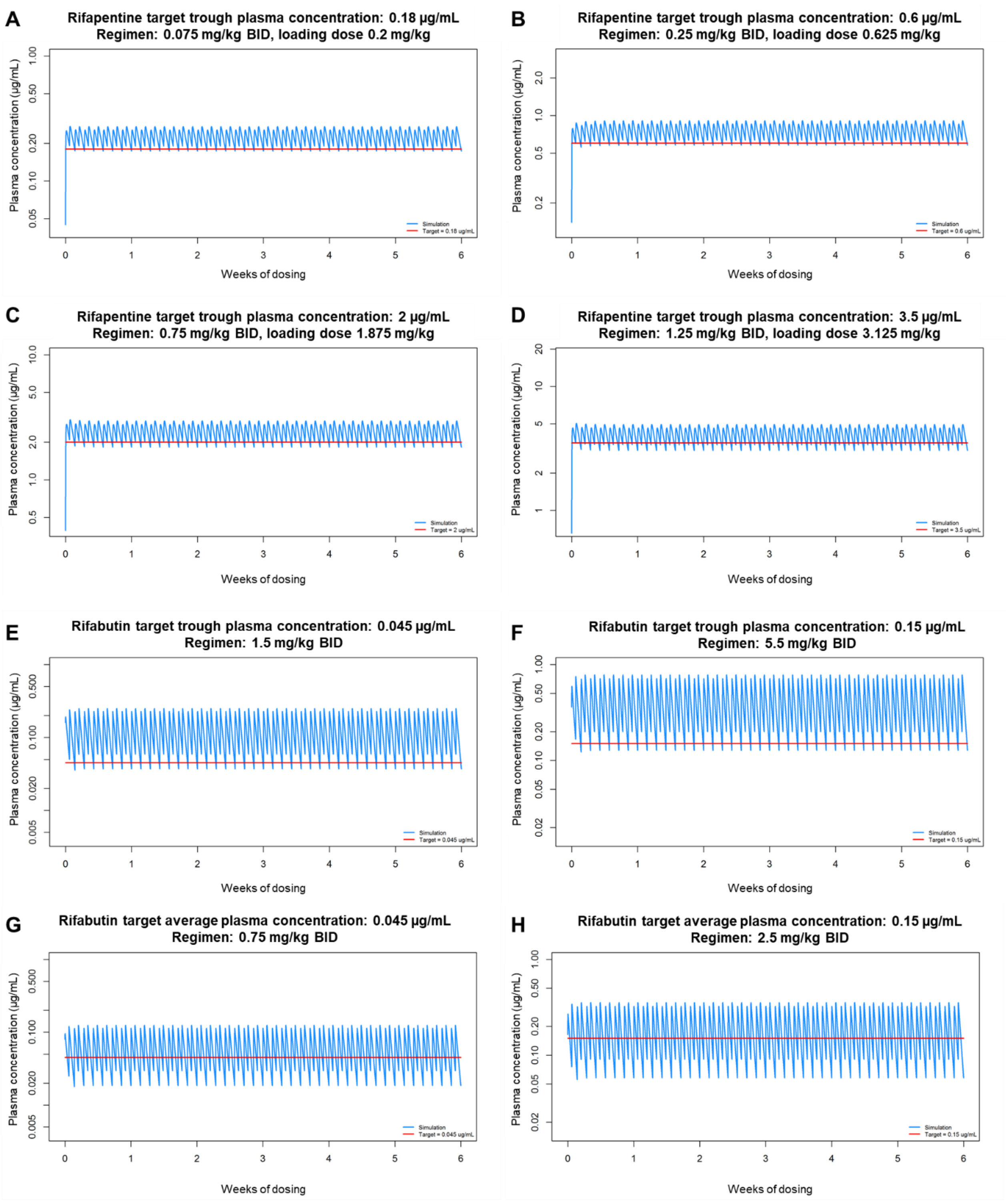

Fig. S2. Dosing simulations for rifapentine (A-D) and rifabutin (E-H) to achieve indicated target plasma concentrations in the first PK/PD study. For rifapentine, regimens were designed to maintain trough plasma concentrations at 0.18, 0.6, 2, and 3.5 µg/mL (panels A-D, respectively). Each rifapentine regimen included a single loading dose, which was the first dose administered when treatment started on Day 0. The Day 10 PK data for rifapentine were not yet available and thus were not incorporated into the PK model used for these simulations. For rifabutin, regimens were designed to maintain trough plasma concentrations at 0.045 µg/mL and 0.15 µg/mL (panels E and F, respectively). Due to the high peak-to-trough predicted plasma exposures, regimens were also designed to maintain average plasma concentrations at 0.045 µg/mL and 0.15 µg/mL (panels G and H, respectively). In all panels, the target plasma concentration is indicated in red, and the simulated drug exposures are indicated in blue. For BID (bis in die, twice daily) dosing, doses were administered 10 and 14 hours apart (7am and 5 pm daily)

A

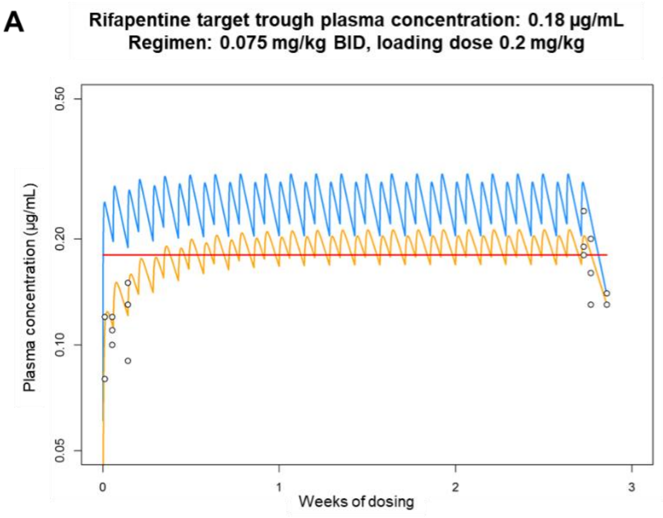

	CL/F (L/h/kg)	V/F (L/kg)	Ka (h^{-1})
param_est	0.03	1.47	0.89
%RSE	4.78	8.58	34.44

B

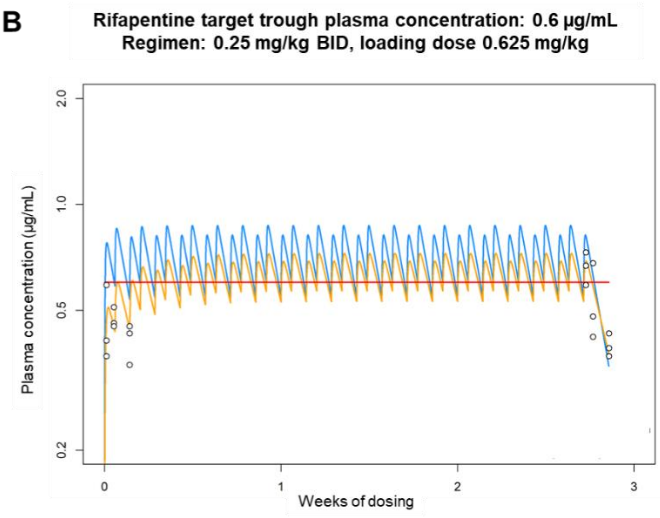

	CL/F (L/h/kg)	V/F (L/kg)	Ka (h^{-1})
param_est	0.03	1.10	1.01
%RSE	4.37	8.73	39.06

C

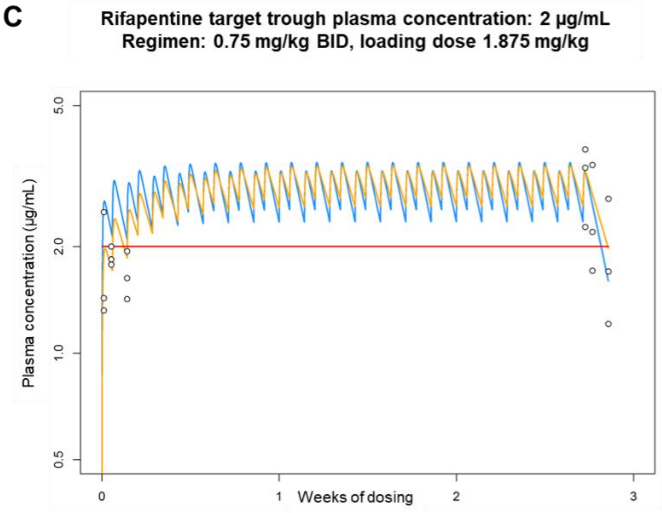

	CL/F (L/h/kg)	V/F (L/kg)	Ka (h^{-1})
param_est	0.02	0.88	1.33
%RSE	7.55	13.70	99.54

D

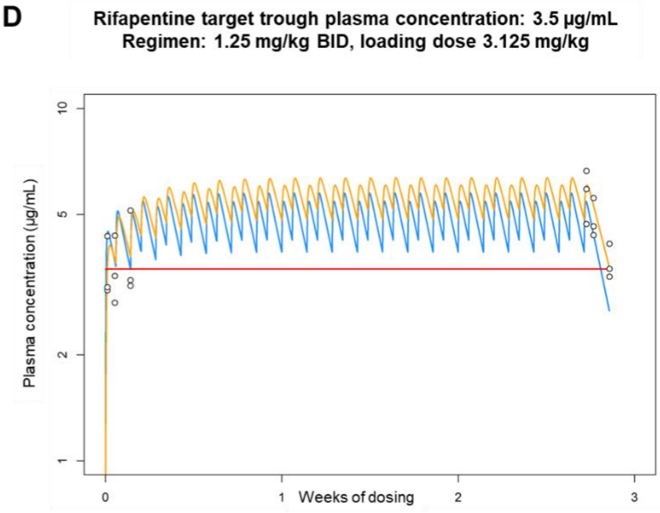

	CL/F (L/h/kg)	V/F (L/kg)	Ka (h^{-1})
param_est	0.02	0.68	0.81
%RSE	5.13	9.90	34.93

Fig. S3. Simulated and observed rifapentine plasma concentrations and PK parameters from the first PK/PD study. Data for regimens with trough plasma target concentrations of 0.18, 0.6, 2, and 3.5 µg/mL are presented in Panels A-D, respectively. The red line indicates the plasma target concentration. Open circles represent the observed plasma concentrations from individual samples. The blue line represents the simulated plasma exposures (based on simulations from model fittings to pilot PK data in uninfected mice, including Day 10 PK data), and the yellow line represents the model fitting to the observed plasma exposures. The lower limit of quantification for rifapentine was 0.10 µg/mL. Parameter estimates for apparent clearance (CL/F), apparent volume of distribution (V/F), and the absorption rate constant (Ka), and the associated percent relative standard error (%RSE) for each regimen are provided below each graph. All individual mouse PK data are provided in **Data File S2**.

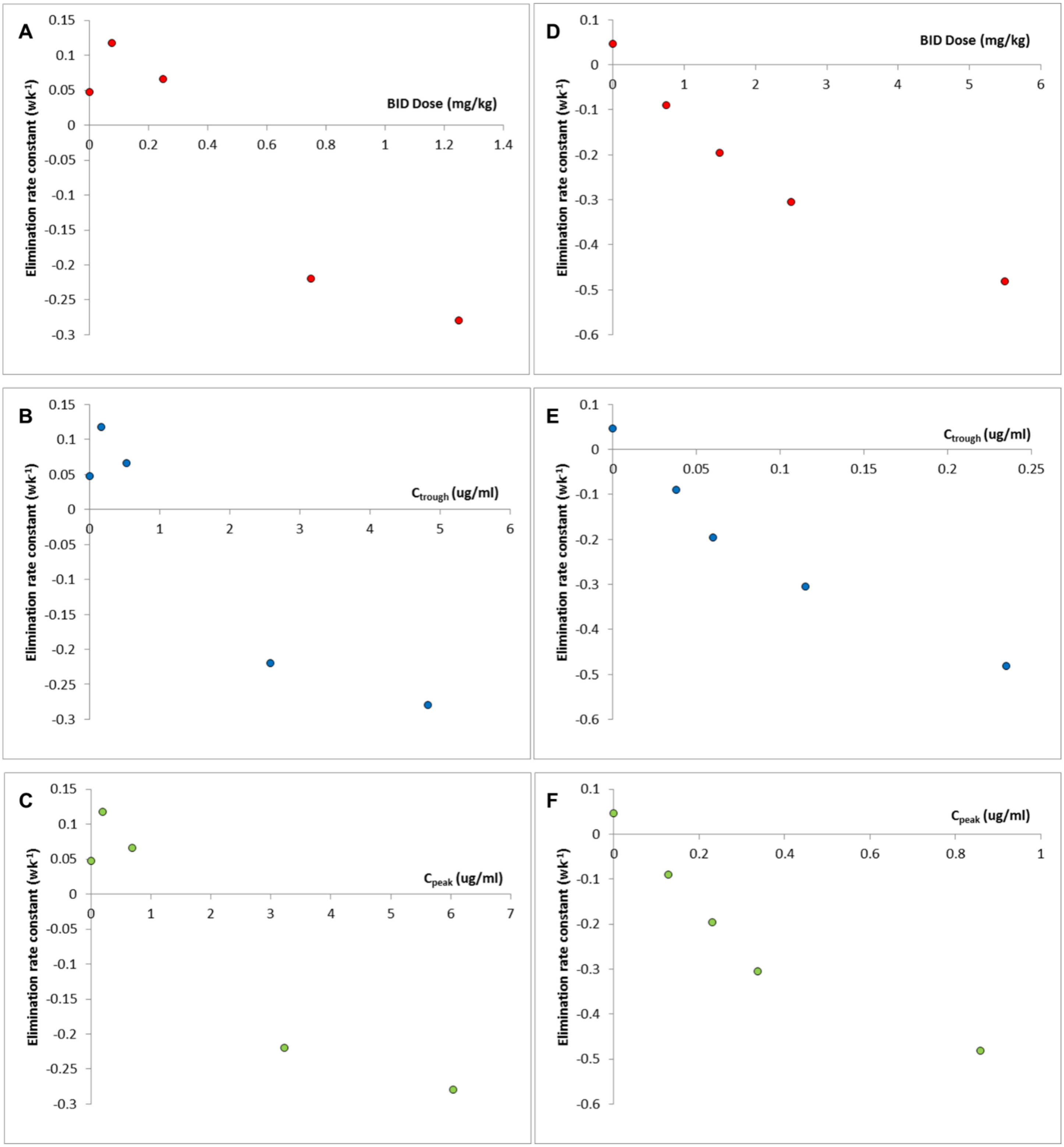

Fig. S4. PK/PD relationships of rifapentine (A-C) and rifabutin (D-F) in the first PK/PD study. For each drug, the bacterial elimination rate constant (K_{net}) versus dose, observed trough plasma concentration (C_{trough}), and observed peak plasma concentration (C_{peak}) are plotted in panels A and D, B and E, and C and F, respectively.

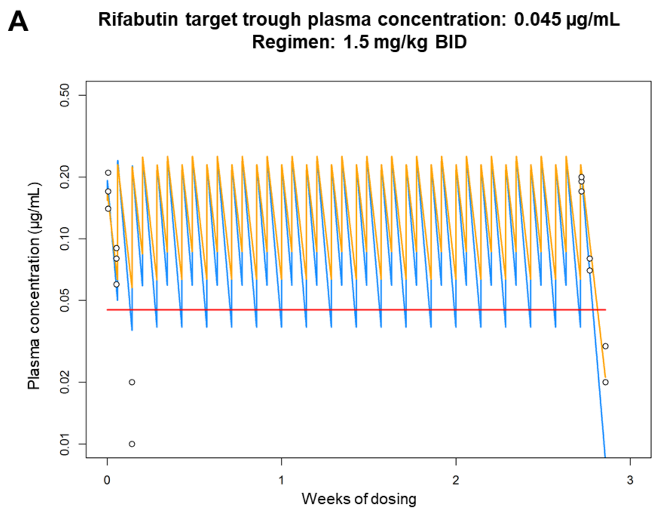

	CL/F (L/h/kg)	V/F (L/kg)	Ka (h^{-1})
param_est	0.88	8.74	23.40
%RSE	9.67	16.01	0.01

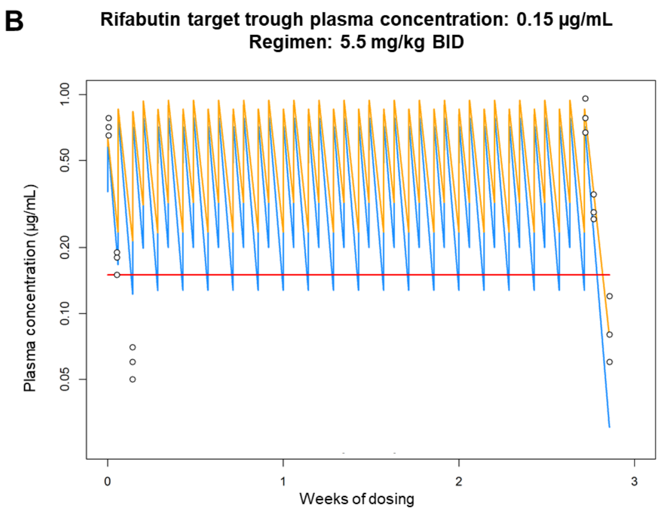

	CL/F (L/h/kg)	V/F (L/kg)	Ka (h^{-1})
param_est	0.86	8.56	24.09
%RSE	10.57	17.49	0.01

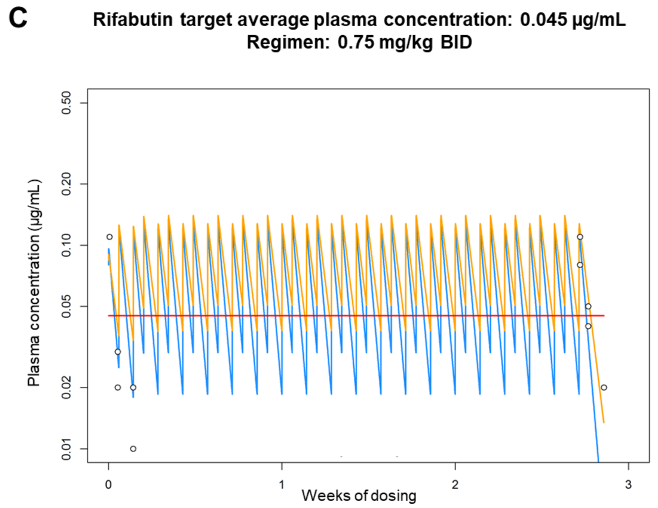

	CL/F (L/h/kg)	V/F (L/kg)	Ka (h^{-1})
param_est	0.77	8.12	25.33
%RSE	10.55	18.25	0.01

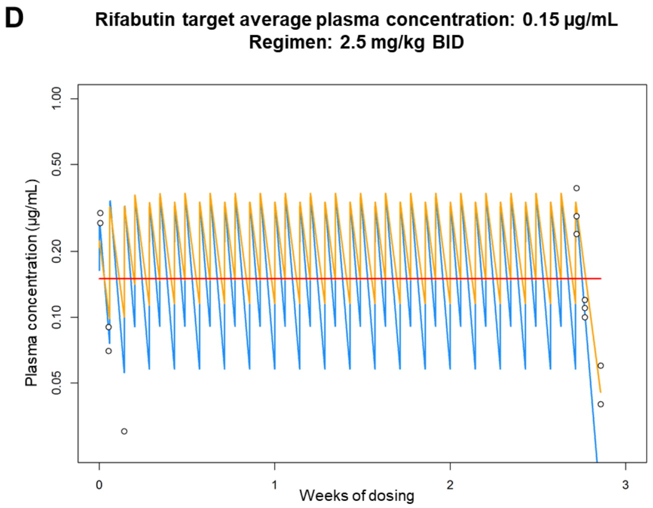

	CL/F (L/h/kg)	V/F (L/kg)	Ka (h^{-1})
param_est	0.93	10.97	25.23
%RSE	11.98	21.50	0.01

Fig. S5. Simulated and observed rifabutin plasma concentrations and PK parameters from the first PK/PD study. Data for regimens with trough plasma target concentrations of 0.045 µg/mL and 0.15 µg/mL are presented in Panels A and B, respectively; data for regimens with average plasma target concentrations of 0.045 µg/mL and 0.15 µg/mL are presented in Panels C and D, respectively. The red line indicates the plasma target concentration. Open circles represent the observed plasma concentrations from individual samples. The blue line represents the simulated plasma exposures, and the yellow line represents the model fitted to the observed plasma exposures. The lower limit of quantification for rifabutin was 0.05 µg/mL. Parameter estimates for apparent clearance (CL/F), apparent volume of distribution (V/F), and the absorption rate constant (Ka), and the associated percent relative standard error (%RSE) for each regimen are provided below each graph. All individual mouse PK data are provided in **Data File S2**.

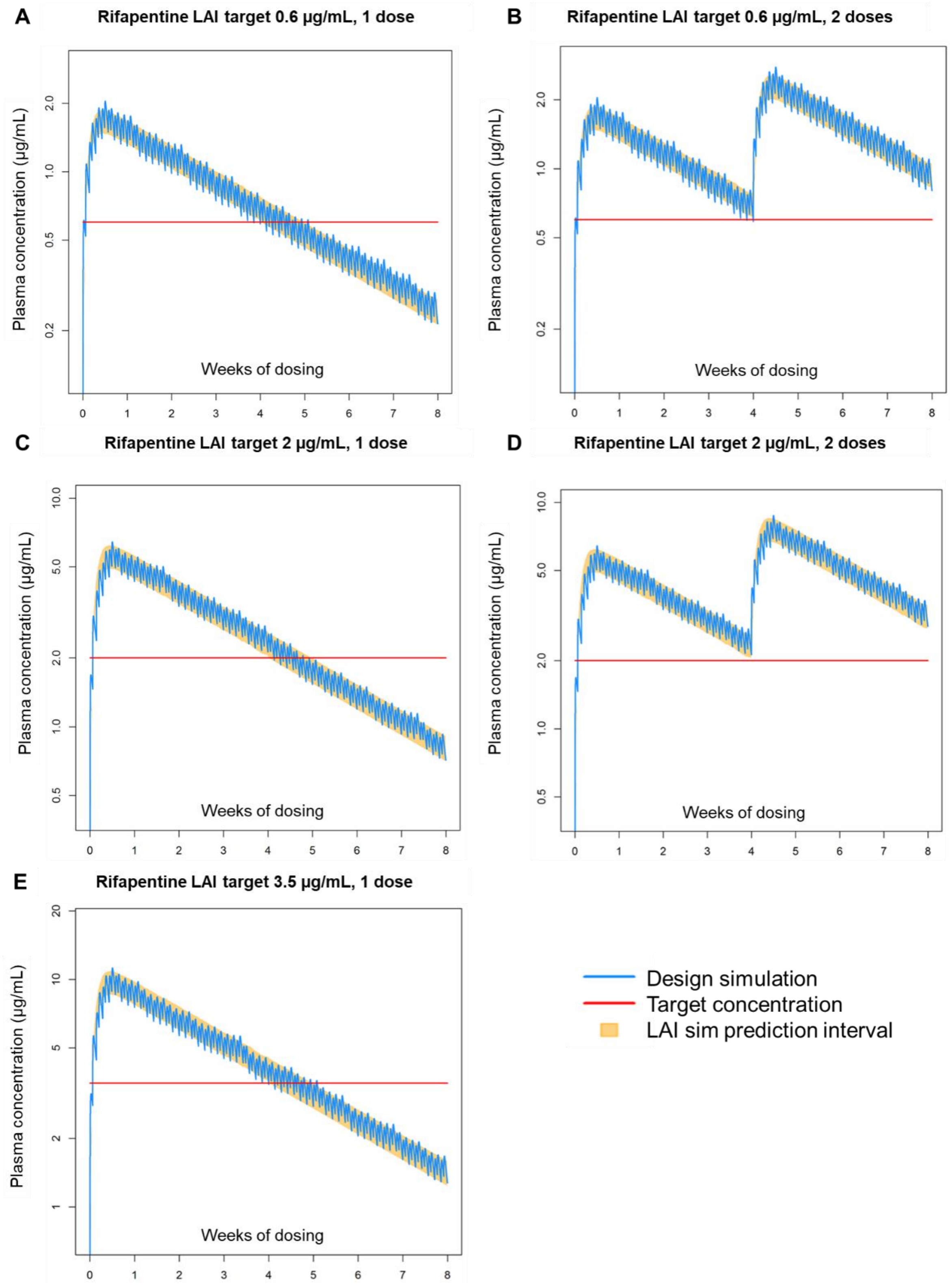

Fig. S6. Exposure profiles and dosing simulations for rifapentine in the second PK/PD study. The red line indicates the target plasma concentration 4 weeks after each simulated LAI dose, and yellow shading indicates the desired exposure profile of a simulated LAI dose of rifapentine. The blue line represents the predicted plasma exposures for the orally-dosed regimen designed to achieve the desired exposure profile. An overview of each regimen is presented in **Table 4**, with detailed descriptions of the dosing provided in **Table S5**. Exposure profiles for a single simulated LAI dose administered at Day 0 are shown in Panels A, C, and E. Exposure profiles for two simulated LAI doses administered at Day 0 and at Week 4 are shown in Panels B and D.

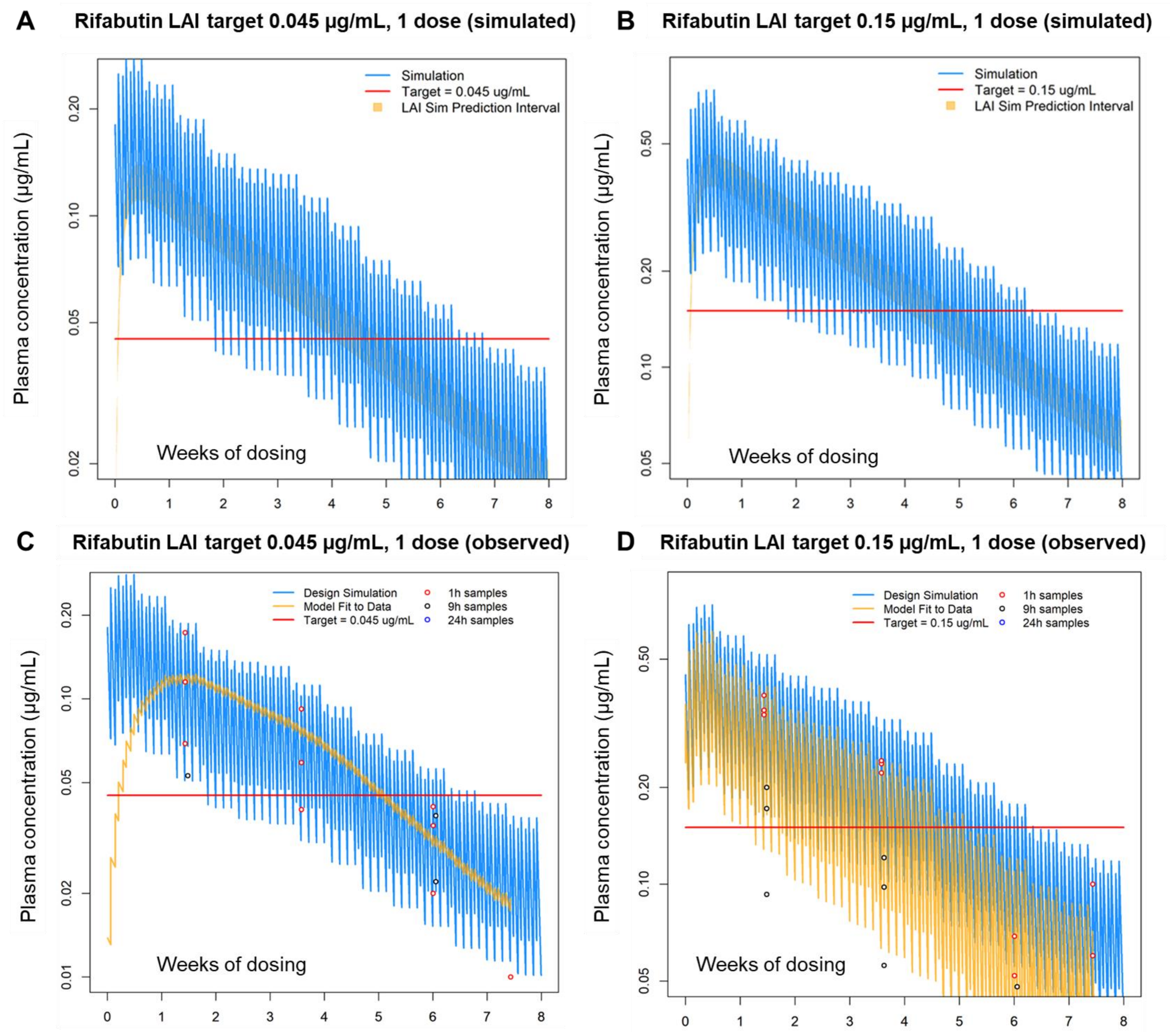

Fig. S7. Rifabutin exposure profiles and dosing simulations (A-B), and observed exposures (C-D) in the second PK/PD study. In all panels, the red line indicates the plasma target concentration, and the blue line represents the predicted plasma exposures for the orally-dosed regimen designed to achieve the desired exposure profile. In Panels A and B, yellow shading indicates the desired exposure profile of a simulated LAI dose of rifabutin. Each exposure profile represents a single simulated LAI dose administered at Day 0. An overview of each regimen is presented in **Table 4**, with detailed descriptions of the dosing provided in **Table S5**. In Panels C and D, the open circles represent the observed plasma concentrations, and the yellow lines represent the modeled exposures based on fitting to the observed data. Modeling of rifabutin exposures was limited because rifabutin was often not detected in mouse plasma at the 9 and 24 hour sampling time points. The lower limit of quantification for rifabutin was 0.05 µg/mL. All individual mouse PK data are provided in **Data File S3**.

Table S1. MIC/MBC data for rifampin, rifapentine, and rifabutin for *M. tuberculosis* H37Rv. Samples were cultured on nonselective 7H11 agar, and culture volume was 500 µL per agar plate.

Replicate and time point	Drug and MIC/MBC results	Concentration (µg/mL)	Visual growth	CFU counts on the following 10-fold dilutions: (shaded cell used to calculate CFU/mL)						CFU/mL	Log_{10} CFU/mL	LLOD (Log_{10} CFU/mL)
				1	2	3	4	5	6			
Replicate 1, Day 0	None (inoculum)	na	0	---	+	+	68	5		1 360 000	6.13	2.30
Replicate 1, Day 14	None (DMSO control)	na	3+	---	---	---	---	---	---	---	---	---
	**Rifampin**<br><br>Results<br>MIC (visual): 0.125 µg/mL<br>MIC (CFUs): ≤0.0625 µg/mL<br>MBC: 0.125 µg/mL	0.0625	1+	---	---	17	0	0	0	34 000	4.53	3.30
		0.125	0	140	13	1	0	---	---	2 600	3.42	1.32
		0.25	0	36	5	0	0	---	---	720	2.86	1.32
		0.5	0	23	3	0	0	---	---	460	2.66	1.32
		1	0	8	2	0	0	---	---	160	2.21	1.32
		2	0	1	0	0	0	---	---	20	1.32	1.32
		4	0	11	4	0	0	---	---	220	2.34	1.32
	**Rifapentine**<br><br>Results<br>MIC (visual): 0.0625 µg/mL<br>MIC (CFUs): ≤0.03125 µg/mL<br>MBC: 0.0625 µg/mL	0.015625	2+	---	---	---	---	---	---	---	---	---
		0.03125	1+	---	---	201	2	1	0	402 000	5.60	3.30
		0.0625	0	108	13	3	0	---	---	2 160	3.33	1.32
		0.125	0	98	25	3	0	---	---	1 960	3.29	1.32
		0.25	0	73	13	1	0	---	---	1 460	3.16	1.32
		0.5	0	20	1	0	0	---	---	400	2.60	1.32
		1	0	2	0	0	0	---	---	40	1.61	1.32
	**Rifabutin**<br><br>Results<br>MIC (visual): 0.0156 µg/mL<br>MIC (CFUs): ≤0.0078 µg/mL<br>MBC: 0.0156 µg/mL	0.000976563	3+	---	---	---	---	---	---	---	---	---
		0.001953125	3+	---	---	---	---	---	---	---	---	---
		0.00390625	2+	---	---	---	---	---	---	---	---	---
		0.0078125	1+	---	---	92	16	2	0	184 000	5.26	3.30
		0.015625	0	91	14	2	0	---	---	1 820	3.26	1.32
		0.03125	0	89	15	2	0	---	---	1 780	3.25	1.32
		0.0625	0	61	5	0	0	---	---	1 220	3.09	1.32
		0.125	0	12	0	0	0	---	---	240	2.38	1.32
		0.25	0	0	0	0	0	---	---	0	0.00	1.32
		0.5	0	0	0	0	0	---	---	0	0.00	1.32
Replicate 2, Day 0	None (inoculum)	na	0	+	+	+	64	3	---	1 280 000	6.11	1.32
Replicate 2, Day 14	None (DMSO control)	na	3+	---	---	---	---	---	---	---	---	---
	**Rifampin**<br><br>Results<br>MIC (visual): 0.25 µg/mL<br>MIC (CFUs): ≤0.125 µg/mL<br>MBC: 0.25 µg/mL	0.0625	2+	---	---	---	---	---	---	---	---	---
		0.125	1+	---	---	43	5	0	0	86 000	4.93	3.30
		0.25	0	300	13	0	0	---	---	2 600	3.42	1.32
		0.5	0	1	0	0	0	---	---	20	1.32	1.32
		1	0	1	0	0	0	---	---	20	1.32	1.32
		2	0	0	0	0	0	---	---	0	0.00	1.32
		4	0	0	0	0	0	---	---	0	0.00	1.32
	**Rifapentine**<br><br>Results<br>MIC (visual): 0.0625 µg/mL<br>MIC (CFUs): ≤0.03125 µg/mL<br>MBC: 0.0625 µg/mL	0.015625	2+	---	---	---	---	---	---	---	---	---
		0.03125	1+	---	---	70	3	0	0	140 000	5.15	3.30
		0.0625	0	300	5	0	0	---	---	1 000	3.00	1.32
		0.125	0	2	0	0	0	---	---	40	1.61	1.32
		0.25	0	3	0	0	0	---	---	60	1.79	1.32
		0.5	0	0	0	0	0	---	---	0	0.00	1.32
		1	0	0	0	0	0	---	---	0	0.00	1.32
	**Rifabutin**<br><br>Results<br>MIC (visual): 0.0156 µg/mL<br>MIC (CFUs): ≤0.0078 µg/mL<br>MBC: 0.0156 µg/mL	0.000976563	3+	---	---	---	---	---	---	---	---	---
		0.001953125	3+	---	---	---	---	---	---	---	---	---
		0.00390625	2+	---	---	---	---	---	---	---	---	---
		0.0078125	1+	---	---	130	14	0	0	260 000	5.41	3.30
		0.015625	0	300	26	3	0	---	---	5 200	3.72	1.32
		0.03125	0	4	1	0	0	---	---	80	1.91	1.32
		0.0625	0	3	0	0	0	---	---	60	1.79	1.32
		0.125	0	0	0	0	0	---	---	0	0.00	1.32
		0.25	0	0	0	0	0	---	---	0	0.00	1.32
		0.5	0	0	0	0	0	---	---	0	0.00	1.32

Visual growth recorded as none (0), or 1+, 2+, or 3+ based relative size of visible bacterial pellet.
For CFU counts, + indicates too many colonies to accurately count.
--- indicates not determined; for samples with 2+ or 3+ visual growth, bacterial clumping precluded CFU determination.
CFU/mL (*x*) was log-transformed as $\log_{10}(x+1)$.
LLOD, lower limit of detection.
na, not applicable.
MIC (visual) = lowest concentration that resulted in no visible bacterial growth to the naked eye on Day 14.
MIC (CFUs) = lowest concentration that resulted in no bacterial growth based on Day 14 CFU counts, compared to Day 0.
MBC = lowest concentration that resulted in ≥2 $\log_{10}$ CFU/mL decrease on Day 14 compared to Day 0.

Table S2. PK parameters associated with oral dosing of rifapentine and rifabutin in uninfected male BALB/c mice. All individual mouse PK data are provided in **Data File S1**.

Drug	Dose (mg/kg)	Sampling day	T_{max} (h post dose)	C_{max} (µg/mL)	AUC_{0-24h} (µg.h/mL)	CL/F (L/kg) [% RSE]	V/F (L/kg) [% RSE]	Ka (h^{-1}) [% RSE]	$t_{½}$ (h)
Rifapentine	0.3	0	2	0.491	6.7	0.020 [10.2]	0.449 [17.9]	1.05 [38.9]	15.6
		10	1	0.768	10.5				
	3	0	4	10.6	118.2	0.015 [8.6]	0.287 [17.1]	0.75 [32.0]	13.3
		10	9	7.87	159.8				
Rifabutin	0.3	0	0.5	0.034	0.2	1.39 [6.0]	9.87 [7.1]	48.9 [0.0]	4.9
		10	0.5	0.027	0.2				
	3	0	0.5	0.299	1.8	1.79 [4.0]	11.0 [5.4]	47.7 [0.0]	4.3
		10	1	0.243	1.6				

Day 0 indicates the first day of drug administration (*i.e.*, the first dose).
T_{max}, time point of maximum observed plasma concentration.
C_{max}, maximum observed plasma concentration.
AUC_{0-24h}, area under the plasma concentration versus time curve from 0-24 hours post-dose.
CL/F, apparent clearance.
V/F, apparent volume of distribution.
Ka, absorption rate constant.
$t_{½}$, plasma half-life.
RSE, relative standard error.

Table S3. Original and final experiment schemes for the first PK/PD study evaluating rifapentine (RPT) and rifabutin (RFB) regimens designed to maintain stable, predefined target plasma drug concentrations. The top portion of the table, shaded in gray, represents the original experiment design, and the bottom portion of the table represents the final experiment design. For RPT and RFB regimens, the regimen description indicates the plasma target concentration the regimen was designed to achieve/maintain during treatment. RIF, rifampin. QD, quaque die (once daily). BID, bis in die (twice daily).

	Regimen description	Oral dosing	Number of BALB/c mice sacrificed for lung CFU counts at the following time points:						Total mice
			**rBCG30 immunization**	***M.tb.* Challenge**		**Treatment initiation**	**During treatment**		
			**Week -12**	**Week -6**		**Day 0**	**Week 3**	**Week 6**	
**Original study scheme**	Negative control	Untreated	5	5		5	5	5	25
	Positive control	RIF 10 mg/kg QD					5	5	10
	RPT target C_{trough} 0.18 µg/mL	RPT 0.075 mg/kg BID (0.2 mg/kg load)					5	5	10
	RPT target C_{trough} 0.6 µg/mL	RPT 0.25 mg/kg BID (0.625 mg/kg load)					5	5	10
	RPT target C_{trough} 2 µg/mL	RPT 0.75 mg/kg BID (1.875 mg/kg load)					5	5	10
	RPT target C_{trough} 3.5 µg/mL	RPT 1.25 mg/kg BID (3.125 mg/kg load)					5	5	10
	RFB target C_{ave} 0.045 µg/mL	RFB 0.75 mg/kg BID					5	5	10
	RFB target C_{trough} 0.045 µg/mL	RFB 1.5 mg/kg BID					5	5	10
	RFB target C_{ave} 0.15 µg/mL	RFB 2.5 mg/kg BID					5	5	10
	RFB target C_{trough} 0.15 µg/mL	RFB 5.5 mg/kg BID					5	5	10
	**TOTAL MICE**		**5**	**5**	**0**	**5**	**50**	**50**	**115***

	Regimen description	Oral dosing	Number of BALB/c mice sacrificed for lung CFU counts at the following time points:						Total mice
			**rBCG30 immunization**	***M.tb.* challenge 1**	***M.tb.* challenge 2**	**Treatment initiation**	**During treatment**		
			**Week -19**	**Week -13**	**Week -7**	**Day 0**	**Week 3**	**Week 3 + 1.5 days**	
**Final study scheme**	Negative control	Untreated	5	5	3 previously infected + 5 new**	5	5	5	33
	Positive control	RIF 10 mg/kg QD					5	5	10
	RPT target C_{trough} 0.18 µg/mL	RPT 0.075 mg/kg BID (0.2 mg/kg load)					5	6	11
	RPT target C_{trough} 0.6 µg/mL	RPT 0.25 mg/kg BID (0.625 mg/kg load)					5	5	10
	RPT target C_{trough} 2 µg/mL	RPT 0.75 mg/kg BID (1.875 mg/kg load)					5	5	10
	RPT target C_{trough} 3.5 µg/mL	RPT 1.25 mg/kg BID (3.125 mg/kg load)					5	5	10
	RFB target C_{ave} 0.045 µg/mL	RFB 0.75 mg/kg BID					5	6	11
	RFB target C_{trough} 0.045 µg/mL	RFB 1.5 mg/kg BID					5	5	10
	RFB target C_{ave} 0.15 µg/mL	RFB 2.5 mg/kg BID					5	5***	10
	RFB target C_{trough} 0.15 µg/mL	RFB 5.5 mg/kg BID					5	5	10
	**TOTAL MICE**		**5**	**5**	**8**	**5**	**50**	**52**	**125**

*120 mice were ordered and immunized with rBCG30; the 5 extra mice were allocated as follows in the final study scheme: 3 mice sacrificed at the time of second *M. tuberculosis* challenge infection (Week -6), annotated as previously infected mice; and 1 mouse was added to each of the lowest RPT and RFB dosing regimens.

**Five new, previously uninfected mice were included in the second *M. tuberculosis* challenge infection and were sacrificed the day after infection to determine the implantation associated specifically with the second infection.

***One mouse died during blood sampling on Day 0; therefore, CFU data are only available for 4 mice in this group.

Table S4. Summary of *M. tuberculosis* H37Rv lung CFU counts in first PK/PD study. "Week 3 (all)" represents the combination of data from the Week 3 and Week 3 + 1.5 days time points. Data represent the mean (standard deviation) for each regimen at each time point. Unless otherwise noted, n = 5 mice per group per time point. See study scheme in **Table S3**. Individual mouse CFU data are presented in **Data S2**.

Regimen description	Oral dosing	*M. tuberculosis* H37Rv mean (SD) log_{10} CFU/lung at the following time points:						
		rBCG30 immunization	*M.tb.* challenge 1	*M.tb.* challenge 2	Treatment initiation	During treatment		
		Week -19	Week -13	Week -7	Day 0	Week 3	Week 3 + 1.5 days	Week 3 (all)
Negative control	Untreated	N/A	0.48 (0.29)	Previously infected: 3.23 (0.72)<br>Newly infected: 1.78 (0.04)*	4.47 (0.52)	4.57 (0.19)	4.62 (0.15)	4.59 (0.16)
Positive control	RIF 10 mg/kg QD					3.85 (0.40)	3.76 (0.17)	3.81 (0.29)
RPT target C_{trough} 0.18 µg/mL	RPT 0.075 mg/kg BID (0.2 mg/kg load)					4.93 (0.38)	4.73 (0.15)	4.82 (0.28)
RPT target C_{trough} 0.6 µg/mL	RPT 0.25 mg/kg BID (0.625 mg/kg load)					4.53 (0.27)	4.77 (0.18)	4.65 (0.25)
RPT target C_{trough} 2 µg/mL	RPT 0.75 mg/kg BID (1.875 mg/kg load)					3.66 (0.18)	3.87 (0.23)	3.77 (0.23)
RPT target C_{trough} 3.5 µg/mL	RPT 1.25 mg/kg BID (3.125 mg/kg load)					3.70 (0.29)	3.50 (0.17)	3.60 (0.25)
RFB target C_{ave} 0.045 µg/mL	RFB 0.75 mg/kg BID					4.48 (0.20)	3.95 (0.41)	4.19 (0.42)
RFB target C_{trough} 0.045 µg/mL	RFB 1.5 mg/kg BID					3.99 (0.29)	3.73 (0.42)	3.86 (0.37)
RFB target C_{ave} 0.15 µg/mL	RFB 2.5 mg/kg BID					3.52 (0.45)	3.51 (0.21)**	3.51 (0.34)
RFB target C_{trough} 0.15 µg/mL	RFB 5.5 mg/kg BID					2.91 (0.21)	3.03 (0.47)	2.97 (0.35)

*Previously infected mice, n = 3; newly infected mice, n = 5.
**n = 4 mice.
N/A, not applicable.

Table S5. Oral dosing regimens designed to simulate LAI exposures of rifapentine and rifabutin in the second PK/PD study. Regimens were administered for a total of 8 weeks, which was divided into 14 four-day dosing periods. Gray and blue shading indicate dosing during Weeks 1-4 and Weeks 5-8 of the study, respectively. For each simulated LAI regimen, the indicated dose was administered twice daily. For the 1HP positive control regimen (not in table), rifapentine at 10 mg/kg was dosed 1 hour before isoniazid at 10 mg/kg. All regimens were administered 7 days/week.

Dosing period	Oral rifapentine dose (mg/kg) for each of the following regimens:					Oral rifabutin dose (mg/kg) for each of the following regimens:	
	LAI target 0.6 µg/mL 1 dose	LAI target 0.6 µg/mL 2 doses	LAI target 2 µg/mL 1 dose	LAI target 2 µg/mL 2 doses	LAI target 3.5 µg/mL 1 dose	LAI target 0.045 µg/mL 1 dose	LAI target 0.15 µg/mL 1 dose
Period 1 (Days 0-3)	0.75	0.75	1.6	1.6	2.4	1.5	5
Period 2 (Days 4-7)	0.6	0.6	1.2	1.2	1.9	1.25	4
Period 3 (Days 8-11)	0.5	0.5	1.1	1.1	1.6	1	3.5
Period 4 (Days 12-15)	0.45	0.45	0.9	0.9	1.4	0.8	3
Period 5 (Days 16-19)	0.375	0.375	0.8	0.8	1.2	0.725	2.75
Period 6 (Days 20-23)	0.325	0.325	0.7	0.7	1.1	0.7	2.5
Period 7 (Days 24-27)	0.275	0.275	0.6	0.6	0.875	0.6	2.2
Period 8 (Days 28-31)	0.24	1	0.5	2.1	0.8	0.5	2
Period 9 (Days 32-35)	0.21	0.8	0.425	1.625	0.7	0.4	1.6
Period 10 (Days 36-39)	0.18	0.7	0.375	1.475	0.6	0.35	1.4
Period 11 (Days 40-43)	0.16	0.6	0.325	1.225	0.5	0.3	1.2
Period 12 (Days 44-47)	0.14	0.52	0.275	1.075	0.45	0.25	1
Period 13 (Days 48-51)	0.12	0.45	0.25	0.95	0.375	0.225	0.9
Period 14 (Days 52-55)	0.1	0.375	0.2	0.8	0.325	0.2	0.8

Table S6. Experiment scheme for the second PK/PD study evaluating simulated LAI regimens of orally dosed rifapentine and rifabutin. The regimens are summarized in **Table 4**, with detailed descriptions of the dosing provided in **Table S5**.

**Regimen description**	**Number of BALB/c mice sacrificed for lung CFU counts at the following time points:**						**Total mice**
	**rBCG30 immunization**	***M.tb.* Challenge**	**Treatment initiation**	**During treatment**			
	**Week -12**	**Week -6**	**Day 0**	**Week 2**	**Week 4**	**Week 8**	
Negative control	8	16*	8	5	5	5	47
Positive control: 1HP				5	5	5	15
RPT LAI target 0.6 µg/mL, 1 dose				5	5	5	15
RPT LAI target 0.6 µg/mL, 2 doses						5	5
RPT LAI target 2 µg/mL, 1 dose				5	5	5	15
RPT LAI target 2 µg/mL, 2 doses						5	5
RPT LAI target 3.5 µg/mL, 1 dose				5	5	5	15
RFB LAI target 0.045 µg/mL, 1 dose				5	5	5	15
RFB LAI target 0.15 µg/mL, 1 dose				5	5	5	15
**TOTAL MICE**	**8**	**16**	**8**	**35**	**35**	**45**	**147**

*Includes 8 mice that were not previously infected with rBCG30.

Table S7. Summary of *M. tuberculosis* H37Rv lung CFU counts in second PK/PD study. Data represent the mean (standard deviation) for each regimen at each time point. Unless otherwise noted, n = 5 mice per group per time point. See study scheme in **Table S6**. Individual mouse CFU data are presented in **Data S3**.

Regimen description	*M. tuberculosis* H37Rv mean (SD) $\log_{10}$ CFU/lung at the following time points:					
	rBCG30 immunization	*M.tb.* Challenge	Treatment initiation	During treatment		
	Week -12	Week -6	Day 0	Week 2	Week 4	Week 8
Negative control	N/A	3.29 (0.15)*	5.80 (0.21)*	5.60 (0.21)	5.42 (0.33)	5.60 (0.22)
Positive control: 1HP				5.03 (0.31)	3.27 (0.19)	
RPT LAI target 0.6 µg/mL, 1 dose				5.50 (0.43)	4.49 (0.35)**	4.63 (0.19)
RPT LAI target 0.6 µg/mL, 2 doses						3.58 (0.37)
RPT LAI target 2 µg/mL, 1 dose				5.28 (0.64)	3.95 (0.13)	3.52 (0.19)
RPT LAI target 2 µg/mL, 2 doses						2.74 (0.17)
RPT LAI target 3.5 µg/mL, 1 dose				4.93 (0.13)	3.17 (0.49)	2.48 (0.15)
RFB LAI target 0.045 µg/mL, 1 dose				5.44 (0.32)	5.00 (0.23)	3.40 (0.51)
RFB LAI target 0.15 µg/mL, 1 dose				5.01 (0.32)	3.69 (0.24)	2.38 (0.43)

*n = 8 mice

**Lung homogenate from one mouse was lost during processing; therefore, CFU data are only available for 4 mice in this group.

N/A, not applicable.

Table S8. Description and CFU data for bacterial suspensions used for aerosol infections of mice. Samples were cultured on selective 7H11 agar without selective drugs (plain agar) and selective 7H11 agar supplemented with 40 µg/mL hygromycin (HYG agar); culture volume was 500 µL per agar plate. See PK/PD experiment schemes in **Table S3** (first study) and **Table S6** (second study).

Description		Agar type	CFU counts for the following 10-fold dilutions: (shaded cell used to calculate CFU/mL)						CFU/mL	log_{10} CFU/mL	LLOD (log_{10} CFU/mL)
			0	1	2	3	4	5			
First PK/PD study (stable drug exposures)	**Week -19, *M. bovis* rBCG30 suspension used for immunization infection** Growing culture, OD_{600} = 1.354, diluted in media to OD_{600} = 0.417; this diluted suspension was used for aerosol infection	Plain	+	+	+	+	~120	21	4,200,000	6.62	0.48
	**Week -13, *M. tuberculosis* H37Rv suspension used for first challenge infection** Frozen stock at OD_{600} = 0.9 was thawed and then diluted 5000-fold in PBS; this diluted suspension was used for aerosol infection	Plain	+	+	134	10	1	---	20,000	4.30	0.48
	**Week -7, *M. tuberculosis* H37Rv suspension used for second challenge infection** Frozen stock at OD_{600} = 0.95 was thawed and then diluted 100-fold in PBS; this diluted suspension was used for aerosol infection	Plain	---	+	+	74	7	0	148,000	5.17	1.32
Second PK/PD study (LAI simulations)	**Week -12, *M. bovis* rBCG30 suspension used for immunization infection** Growing culture, OD_{600} = 1.097, diluted in media to OD_{600} = 0.400; this diluted suspension was used for aerosol infection	Plain	---	+	+	+	+	~400	80,000,000	7.90	1.32
		HYG	---	+	+	Contam.	+	~400	80,000,000	7.90	1.32
	**Week -6, *M. tuberculosis* H37Rv suspension used for challenge infection** Frozen stock at OD_{600} = 1.0 was thawed and then diluted 100-fold in PBS; this diluted suspension was used for aerosol infection	Plain	---	+	+	+	~500	51	10,200,000	7.01	1.32
		HYG	---	+	+	+	~500	46	9,200,000	6.96	1.32

Selective agar was used because this agar had been prepared for plating the mouse lung homogenates associated with each infection time point.
For CFU counts, + indicates too many colonies to accurately count; ~ indicates exact CFU count could not be determined due to merged/touching colonies;
--- indicates not determined; and Contam. indicates bacterial of fungal contamination on agar that precluded CFU determination.
CFU/mL (x) was log-transformed as log_{10} (x+1).
LLOD, lower limit of detection.

Table S9. Summary of *M. bovis* rBCG30 lung CFU counts in first PK/PD study. "Week 3 (all)" represents the combination of data from the Week 3 and Week 3 + 1.5 days time points. Data represent the mean (standard deviation) for each regimen at each time point. Unless otherwise noted, n = 5 mice per group per time point. See experiment scheme in **Table S3**. Individual mouse CFU data are presented in **Data File S2**.

Regimen description	Oral dosing	*M. bovis* rBCG30 mean (SD) log_{10} CFU/lung at the following time points:						
		rBCG30 immunization	*M.tb.* challenge 1	*M.tb.* challenge 2	Treatment initiation	During treatment		
		Week -19	Week -13	Week -7	Day 0	Week 3	Week 3 + 1.5 days	Week 3 (all)
Negative control	Untreated	3.19 (0.09)	5.00 (0.18)	3.29 (0.32)*	3.73 (0.50)	3.47 (0.20)	3.23 (0.26)	3.35 (0.25)
Positive control	RIF 10 mg/kg QD					1.14 (0.31)	0.90 (0.54)	1.02 (0.43)
RPT target C_{trough} 0.18 µg/mL	RPT 0.075 mg/kg BID (0.2 mg/kg load)					2.74 (0.73)	2.70 (0.48)	2.72 (0.57)
RPT target C_{trough} 0.6 µg/mL	RPT 0.25 mg/kg BID (0.625 mg/kg load)					1.32 (0.42)	1.42 (0.66)	1.37 (0.52)
RPT target C_{trough} 2 µg/mL	RPT 0.75 mg/kg BID (1.875 mg/kg load)					1.17 (0.22)	0.51 (0.69)	0.84 (0.60)
RPT target C_{trough} 3.5 µg/mL	RPT 1.25 mg/kg BID (3.125 mg/kg load)					0.55 (0.53)	0.33 (0.74)	0.44 (0.62)
RFB target C_{ave} 0.045 µg/mL	RFB 0.75 mg/kg BID					2.49 (0.51)	1.43 (0.71)	1.91 (0.81)
RFB target C_{trough} 0.045 µg/mL	RFB 1.5 mg/kg BID					1.85 (0.31)	1.79 (0.63)	1.82 (0.47)
RFB target C_{ave} 0.15 µg/mL	RFB 2.5 mg/kg BID					1.80 (0.78)	1.46 (0.42)**	1.65 (0.63)
RFB target C_{trough} 0.15 µg/mL	RFB 5.5 mg/kg BID					0.40 (0.56)	0.16 (0.35)	0.28 (0.46)

*n = 3 mice
**n = 4 mice

Table S10. Summary of *M. bovis* rBCG30 lung CFU counts in the second PK/PD study. Data represent the mean (standard deviation) for each regimen at each time point. Unless otherwise noted, n = 5 mice per group per time point. See study scheme in **Table S6**. Individual mouse CFU data are presented in **Data File S3**.

Regimen description	***M. bovis*** **rBCG30 mean (SD) $\log_{10}$ CFU/lung at the following time points:**					
	**rBCG30 immunization**	***M.tb.*** **Challenge**	**Treatment initiation**	**During treatment**		
	**Week -12**	**Week -6**	**Day 0**	**Week 2**	**Week 4**	**Week 8**
Negative control	3.61 (0.06)*	5.70 (0.15)*	3.62 (0.16)*	4.16 (0.20)	3.59 (0.32)	3.41 (0.39)
Positive control: 1HP				1.66 (0.15)	0 (0)	
RPT LAI target 0.6 µg/mL, 1 dose				2.61 (0.39)	1.91 (0.48)**	1.12 (0.25)
RPT LAI target 0.6 µg/mL, 2 doses						0.52 (0.49)
RPT LAI target 2 µg/mL, 1 dose				2.60 (0.67)	1.62 (0.19)	0.92 (0.55)
RPT LAI target 2 µg/mL, 2 doses						0.16 (0.35)
RPT LAI target 3.5 µg/mL, 1 dose				1.94 (0.70)	0.87 (0.55)	0.30 (0.67)
RFB LAI target 0.045 µg/mL, 1 dose				3.01 (0.41)	1.91 (0.27)	0.93 (0.59)
RFB LAI target 0.15 µg/mL, 1 dose				2.22 (0.28)	0.51 (0.69)	0 (0)

*n = 8 mice

**Lung homogenate from one mouse was lost during processing; therefore, CFU data are only available for 4 mice in this group.
